# Drug Checking: A prevention measure for a heterogeneous group with high consumption frequency and polydrug use - evaluation of zurich's drug checking services

**DOI:** 10.1186/1477-7517-8-16

**Published:** 2011-06-10

**Authors:** Ines Hungerbuehler, Alexander Buecheli, Michael Schaub

**Affiliations:** 1 Research Institute for Public Health and Addiction RIPHA, Konradstrasse 32, Postfach, Zurich, CH-8031, Switzerland; 2 City of Zurich, Social Facilities and Operations, Addiction and Drugs, "Streetwork" Youth Advisory Service, Wasserwerkstr. 17, Zurich, CH-8006, Switzerland

## Abstract

**Background:**

The increasing party culture in Zurich presents new challenges, especially regarding the consumption of alcohol and so-called party drugs. Streetwork, the youth advisory service of the city of Zurich, has provided onsite and stationary Drug Checking facilities since 2001 and 2006, respectively. Drug Checking always involves filling out an anonymous questionnaire, which allows the collection of important information about a largely unknown group of users and their consumption patterns.

**Methods:**

The questionnaires assessed sociodemographic characteristics, consumption patterns, Drug Checking experiences, information behavior and social support. The collected data were statistically analyzed by the Research Institute for Public Health and Addiction (RIPHA).

**Results:**

The majority of Drug Checking service patrons were male and between 20 and 35 years old. These patrons reported high lifetime prevalences and high consumption frequencies of legal and illegal substances, and they often reported polydrug use. Aside from tobacco and alcohol, the most consumed drugs during typical party nights were ecstasy, amphetamines, cannabis and cocaine. Party drug consumers using Drug Checking services form a heterogeneous group with respect to sociodemographic characteristics and consumption patterns. Users of the onsite Drug Checking facilities were significantly younger, were less experienced with drug testing, and reported more polydrug use than users of the stationary Drug Checking service.

**Conclusions:**

Drug Checking combined with a consultation appears to be an important harm reduction and prevention measure that reaches a group of consumers with high consumption frequency and polydrug use. Because of the heterogeneity of the target group, different prevention measures must be offered and embedded in an overall local concept.

## Background

Leisure and entertainment play an important role in welfare societies. Due to the relaxation of Switzerland's hospitality laws (changes in closing times) and the mass phenomenon of the burgeoning techno-culture in the mid-90s, the city of Zurich, with its 380,000 inhabitants, has evolved into one of the most significant party metropolises in Europe. As shown in a survey conducted in Zurich 2003, going out, or as colloquially expressed, "partying", was identified as one of the main leisure activities of the city's residents [1]. More than 100 clubs and dance bars attract close to 50,000 festive people from home and abroad every weekend. Music, fashion and the consumption of legal and illegal substances create the context of entertainment [2]. This development poses new problems, especially regarding the consumption of alcohol and so-called party drugs. The term "party drugs" refers to a variety of substances that are used frequently at raves and dance parties [3]. The results from earlier studies have shown much higher levels of drug use among young people who visit nightclubs than among young people in the general population. For example, in a study by Chinet et al. (2007), 42.0% of dance music event attendees were occasional and 6.0% were daily polydrug users in Switzerland. A total of 22.7% reported using ecstasy, and 20.7% reported using cocaine within the last 30 days [4].

Since 2001, Streetwork, the youth advisory service of the city of Zurich, has provided an onsite Drug Checking service, which is offered at different party events ten times per year. Based on positive experiences with onsite Drug Checking facilities, the Drug Information Centre (DIZ), an information and counseling center that includes Drug Checking, was established in 2006. The number of analyzed samples, people reached, and consultations lasting longer than 15 minutes has increased consistently since 2001.

A study by Benschop et al. (2003) showed that pill-checking users exhibit broad consumption experiences with legal and illegal drugs and often consume various substances together [5]. The Drug Checking service includes free substance analysis and consultations or counseling sessions with a social worker from the Streetwork service. Within the consultation, consumption and substance-specific questions can be answered, and individual drug behaviors can be discussed. The transfer of this specific knowledge can be viewed as a pragmatic attempt to minimize or avoid the consumption of potentially harmful substances [6] and, thus, a measure of harm reduction. In terms of detecting and preventing the possibility of developing an addiction in a population that is deemed to be at risk for substance abuse, Drug Checking is also a measure of selective prevention.

As a kind of return service, users are obliged to complete an anonymous questionnaire within the consultation, collecting important information about a group of users that have been largely unknown so far.

After the first two evaluations of the Drug Checking questionnaires were conducted in 2003 and 2005 [3], a third evaluation was conducted in 2010 in cooperation with the Research Institute for Addiction and Health (ISGF).

## Methods

The present evaluation is an exploratory study of Drug Checking users. Between 2001 and June 2010, the onsite laboratory was present on 84 events, and the DIZ was open on 172 days. A total of 7,622 consultations were completed, and 2,055 high-pressure liquid chromatography (HPLC) analyses were performed. At the time the substances were analyzed, the questionnaires were filled out with a professional from Streetwork. The questionnaires contain questions about sociodemographic variables (age, gender, nationality, educational and employment status), going-out behavior, consumption patterns (such as lifetime prevalences, frequency, and consumption during a typical party night), Drug Checking experiences, information behavior, and social support. The assessment of consumption frequency was adapted over time. Until 2007, the consumption frequency was recorded over the previous twelve months, whereas from 2008, it was assessed over the previous 30 days. Furthermore, in 2008, people were not asked what substances they consumed during a *typical *night but during their *last *party night.

By including the results from previous years, trends and developments regarding consumption frequency and polydrug use were revealed.

Polydrug use was defined as the consumption of more than one substance (tobacco excluded) during a typical party night with at least one being an illegal substance, such as cocaine, ecstasy, amphetamines or opiates.

The collected data were analyzed using SPSS statistical software for Windows, release 17 (SPSS Inc, Chicago, IL, USA). Descriptive statistics were calculated for the sample and consumption patterns. Categories were designated for certain variables, such as age. Comparisons of the variables were made with *t-tests *and, in case of categorical variables, with *Chi-Squared *tests. All analyses were performed with two-sided tests, and p ≤ .05 was considered significant.

## Results

### Sociodemographic characteristics

The evaluated random sample consisted of 1,376 (n) persons. Because one person can have up to two samples analyzed, the number of completed questionnaires (1376) is not the same as the number of substances analyzed (2055).

Of the subjects, 21.9% were women, and the average age was 27.8 years. At the time of the survey, the youngest person was 15, and the oldest was 70. The majority was between 20 and 35 years old (71.2%). Approximately 41% of the respondents cited vocational training as their most recently completed education, 17.4% had a tertiary education degree, 6.1% had not completed their compulsory education or had only been to primary school, 58.2% said they employed at the time of the survey, 16.8% were in vocational training, and 19.8% were unemployed.

### Consumption patterns, trends and related problems

Among the users of the Drug Checking services, the most commonly consumed substances during a typical party night in a club were tobacco (49.9%), alcohol (56.5%), ecstasy (49.9%), amphetamines (37.1%), cannabis (36.2%) and cocaine (27.0%). Thus, not surprisingly, most of those interviewed had consumed cannabis (93.9%), ecstasy tablets or MDMA powder (92.7%), cocaine (80.8%) and/or amphetamines (74.8%) at least once in their life.

**Figure 1:**
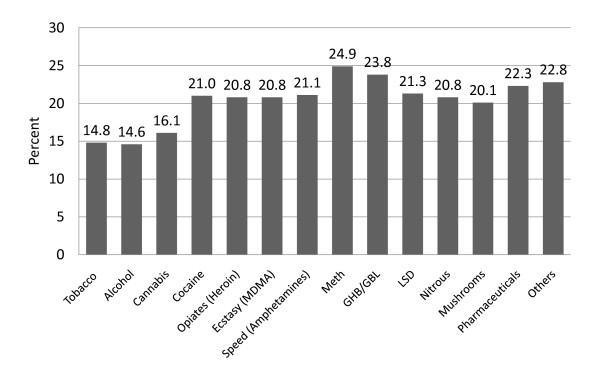
**Initiation age of consumption**. Figure 1 shows the initiation age of consumption of various substances among the evaluated sample of Drug Checking users.

As shown in Figure 1, the initiation age for legal substances (alcohol and tobacco) was approximately 15 and the age for cannabis was approximately 16. Most people were between 20 and 25 years old when they first tried party drugs (e.g., cocaine, opiates, GHB, ecstasy or LSD).

In the analysis of the frequency of consumption, the regular use of cannabis was noteworthy. As shown in Table 1, 27.2% of those interviewed claimed to use cannabis daily, while only 8.6% stated that they drank alcohol on a daily basis. A total of 40.8% of the interviewees reported consuming alcohol once or twice a week, mostly on the weekends. Other substances, such as cocaine, ecstasy, GHB/GBL or amphetamines, were used one to three times per month.

**Table 1:** Frequency of substance use (n = 1376)

	daily (%)	3-6 times/week (%)	1-2 times/week (%)	1-3 times/month (%)
Alcohol	8.6	19.1	40.8	14.3
Cannabis	27.2	8.5	13.8	10.9
Cocaine	2.2	3.8	12.7	17.8
Ecstasy	0.2	0.6	13.1	29.4
Amphetamines	1.1	1.4	12.1	15.7
GHB	0.5	0.5	3.8	4.8
Opiates	0.9	0.1	0.1	0.6
LSD	0.0	0.1	2.0	8.1
Mushrooms	0.1	0.0	0.3	2.0

On average, the weekly consumption of the evaluated sample population increased for alcohol, cannabis and cocaine (2004: 37.2% 11.6% and 10.1%, respectively; 2009: 43.0%, 16.8% and 11.5%, respectively) and decreased for ecstasy and amphetamines (2004: 19.5% and 19.4%, respectively; 2009: 6.3% and 6.2%, respectively) from 2004 to 2009. The monthly consumption of cocaine, ecstasy, amphetamines and GHB increased (2004: 14.0%, 22.7%, 13.2% and 0.8%, respectively; 2009: 22.1%, 39.4%, 23.1% and 5.6%, respectively,). Furthermore, in 2004, 80.3% of the interviewees said that they use tobacco on a daily basis. In 2009, this figure was approximately 24% lower (i.e., 56.4%). In contrast, the number of non-daily smokers increased on average from 5.6% (2004) to 17.2% (2009).

**Figure 2:**
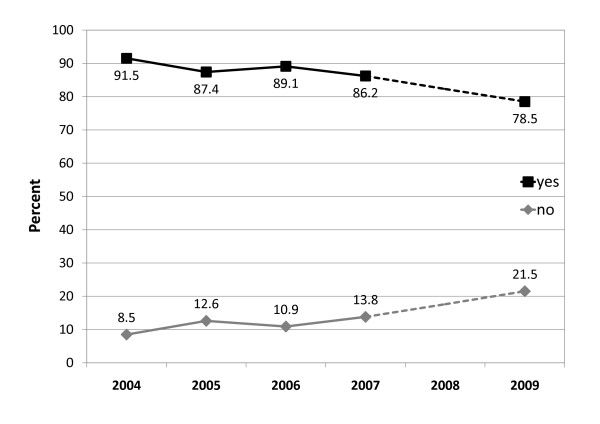
**Polydrug use during a typical party night (n = 1042)**. The majority of the evaluated subjects reported consumption of various substances during a typical party night. Figure 2 shows the trend of polydrug use from 2004 to 2009, which decreased on average by 13.0%.

The majority of the interviewees (81.1%) reported polydrug use during a typical party night. That is, most of the illegal substances, such as cocaine, ecstasy or amphetamines, were consumed together with alcohol and/or cannabis. Nineteen percent of the evaluated sample consumed cocaine together with ecstasy, and 22.2% consumed ecstasy together with amphetamines during a typical party night. The trend shows that polydrug use decreased on average by 13.0% from 2004 to 2009, as shown in Figure 2.

Problems associated with the use of party drugs have been assessed since 2008. A total of 37.6% of the users indicated that they had had a "bad trip". Another 20.9% said that they had suffered from symptoms of depression, and 14.9% had suffered from panic attacks. Another 24.8% had family or relationship problems, and 31.3% had dealings with the legal system.

The comparison between users of the onsite and stationary Drug Checking services showed that the two groups clearly differed with respect to sociodemographic data and consumption patterns. Consumers who used the Drug Checking services in the DIZ were significantly older (30.7 vs. 27.0 years; p = .000), more often female (29.2% vs. 19.8%; p = .001), more often unemployed (30.5% vs. 16.9%; p = .000) and more often had a tertiary educational background (30.8% vs. 16.7%; p = .000) than the onsite Drug Checking users. Furthermore, the users of the DIZ Drug Checking facilities reported more testing experiences (31.4% vs. 23.6%; p = .016) and less polydrug use (76.1% vs. 88.2%; p = .000) than persons who used the onsite Drug Checking facilities. Thus, the consumers who were reached by onsite Drug Checking were significantly younger, were less testing-experienced, and reported more polydrug use than the users of the stationary Drug Checking service.

## Conclusions

For most substances, the regular consumption and lifetime prevalences were much higher for the evaluated sample population than for the general Swiss population (e.g., cannabis 19.4%, ecstasy 1.8% and cocaine 2.8%,) [7] and were even higher than those reported in a study by Chinet et al. (2007), which investigated the substance use habits of dance music event attendees (e.g., cannabis 68.8%, ecstasy 40.4% and cocaine 35.0%) [4]. The results indicate that more drug users report an addictive (daily) consumption of tobacco, alcohol and cannabis than of illegal drugs, such as cocaine, ecstasy or amphetamines. The proportion of those who smoke on a daily basis was 35% higher in the evaluated sample population than Switzerland's average number of smokers in 2009 [8]. The percentage of daily cannabis consumers (27.2%), which was comparable with the results of Chinet et al. (2007), was also clearly higher than in the general Swiss population (9.3% in 2007) [4]. Furthermore, party drugs are often used in combination, particularly with cannabis or alcohol [9]. Accordingly, the majority of participants reported polydrug use during a typical party night.

Taken together, our results show that the target group contains users with high lifetime prevalences, high consumption frequency, polydrug use and negative experiences regarding their consumption. Based on the actual knowledge about the side effects and the long-term effects of recreational drugs, we concluded that the Drug Checking service reaches individuals with high (risky) or even dependent consumption. As shown by the European Pill-testing study [5], Drug Checking is often the first point of contact with the social support system for many users. Facilitating access for this target group through Drug Checking services legitimizes the costs associated with the sophisticated laboratory techniques of substance analyses. Furthermore, by offering these consumers a concrete service (substance analysis), it is easier to motivate them to participate in a consultation or a counseling session. As experience shows, the "obligation" to take part in a counseling session is, for very few individuals, a reason for not analyzing a substance. Additionally, as shown in a study by Benschop et al. (2003), most Drug Checking users rated the counseling that accompanied the testing as highly important [5].

Some limitations to this research merit note. First, when comparing certain variables over time, some adaptations of the questionnaire must be taken into consideration. As already mentioned, in 2008, people were not asked about the substances consumed during a *typical *party night but during their *last *party night. In addition, until 2007, the consumption frequency was recorded over the previous twelve months, whereas from 2008, it was assessed over the previous 30 days. Thus, comparisons of those variables over the years have to be made with caution. Second, consumption in the last 30 days may not be representative of the consumption in a previous period (e.g., last year) and may only reflect current consumption. Third, the quantities of substances consumed and the method of consumption were not assessed. Obviously, the risks associated with drug consumption depend largely on the consumed amount and the method of consumption. Thus, it cannot be clearly determined if a person has participated in risky consumption. Fourth, the quality of substances, in terms of connecting the questionnaire data with the analysis data, was also not included. However, it can be assumed that persons using Drug Checking services are aware of the risk regarding substance quality.

Nevertheless, with the help of these questionnaires, important data have been collected on a group of users that has been largely unknown so far. Thanks to the collection and evaluation of the presented data, the city of Zurich today has a much greater knowledge of the substances used, the consumption patterns, and above all the drug users themselves.

A closer examination of the published literature has shown that each drug has different properties, different users and different consumption settings. The consumer groups differ in terms of age, gender, sexual orientation, and ethnicity [8]. Accordingly, and in contrast to general social opinion, the results of the evaluation of the questionnaire show that the users of party drugs form a quite heterogeneous group, which indicates the need for various measures. For example, the lives of partygoers change as they become older, and party culture becomes less important in their daily lives. Yet, the use of party drugs continues-no longer at parties but increasingly in other settings. Accordingly, persons using the DIZ Drug Checking service are significantly older than those using onsite Drug Checking at parties.

Furthermore, the available results show that this target group can be reached with an acceptance-based approach and that Drug Checking should be embedded in a comprehensive and overall preventative concept. In this way, Drug Checking services provide confidential contact points for the target group, where their issues are critically questioned but also understood. Furthermore, there must be networking and cooperation between the various stakeholders and actors, such as politicians, the police and/or medical-treatment services. For example, thanks to the collaboration of the DIZ with a therapeutic and medical center (GAIN), a connection between a low-threshold institution and a high-threshold institution could be created, and thus, further help (e.g., medical/therapeutic treatment) could be provided when required.

Last but not least, the results indicate that a Drug Checking service combined with a consultation session does not, as some would claim, encourage consumption. As shown, there was no increase either in the frequency of consumption of most party drugs or in polydrug use over the years. This observation is in line with the results of Benschop et al. (2003), who found that information offered within this service even resulted in restricted consumption among ecstasy users [5].

The knowledge developed within the present evaluation will hopefully encourage other party metropolises to create new and improved services or to redefine existing services based on a realistic and acceptance-based drug prevention approach embedded within an overall local concept.

## Competing interests

The authors declare that they have no competing interests.

## Authors' contributions

IH was responsible for the data analyses and prepared the first draft of the paper and the final manuscript. AB assisted with the interpretation of the data, provided the main background content and provided critical comments on the manuscript. MS supervised the data analyses and manuscript preparation and critically revised the final draft. All of the authors approved the final version submitted for publication.
